# Ultrastructural studies of ALS mitochondria connect altered function and permeability with defects of mitophagy and mitochondriogenesis

**DOI:** 10.3389/fncel.2015.00341

**Published:** 2015-09-01

**Authors:** Riccardo Ruffoli, Alessia Bartalucci, Alessandro Frati, Francesco Fornai

**Affiliations:** ^1^Department of Translational Research and New Technologies in Medicine and Surgery, University of PisaPisa, Italy; ^2^I.R.C.C.S., NeuromedPozzilli, Italy

**Keywords:** mitochondria, amyotrophic lateral sclerosis, autophagy, human patients, motor neuron, electron microscopy, biogenesis of mitochondria

## Abstract

The key role of mitochondria in patients affected by amyotrophic lateral sclerosis (ALS) is well documented by electron microscopy studies of motor neurons within spinal cord and brainstem. Nonetheless, recent studies challenged the role of mitochondria placed within the cell body of motor neuron. In fact, it was demonstrated that, despite preservation of mitochondria placed within this compartment, there is no increase in the lifespan of transgenic mouse models of ALS. Thus, the present mini-review comments on morphological findings of mitochondrial alterations in ALS patients in connection with novel findings about mitochondrial dynamics within various compartments of motor neurons. The latter issue was recently investigated in relationship with altered calcium homeostasis and autophagy, which affect mitochondria in ALS. In fact, it was recently indicated that a pathological mitophagy, mitochondriogenesis and calcium homeostasis produce different ultrastructural effects within specific regions of motor neurons. This might explain why specific compartments of motor neurons possess different thresholds to mitochondrial damage. In particular, it appears that motor axons represent the most sensitive compartment which undergoes the earliest and most severe alterations in the course of ALS. It is now evident that altered calcium buffering is compartment-dependent, as well as mitophagy and mitochondriogenesis. On the other hand, mitochondrial homeostasis strongly relies on calcium handling, the removal of altered mitochondria through the autophagy flux (mitophagy) and the biogenesis of novel mitochondria (mitochondriogenesis). Thus, recent findings related to altered calcium storage and impaired autophagy flux in ALS may help to understand the occurrence of mitochondrial alterations as a hallmark in ALS patients. At the same time, the compartmentalization of such dysfunctions may be explained considering the compartments of calcium dynamics and autophagy flux within motor neurons.

## Introductory Statement

**Figure 1:**
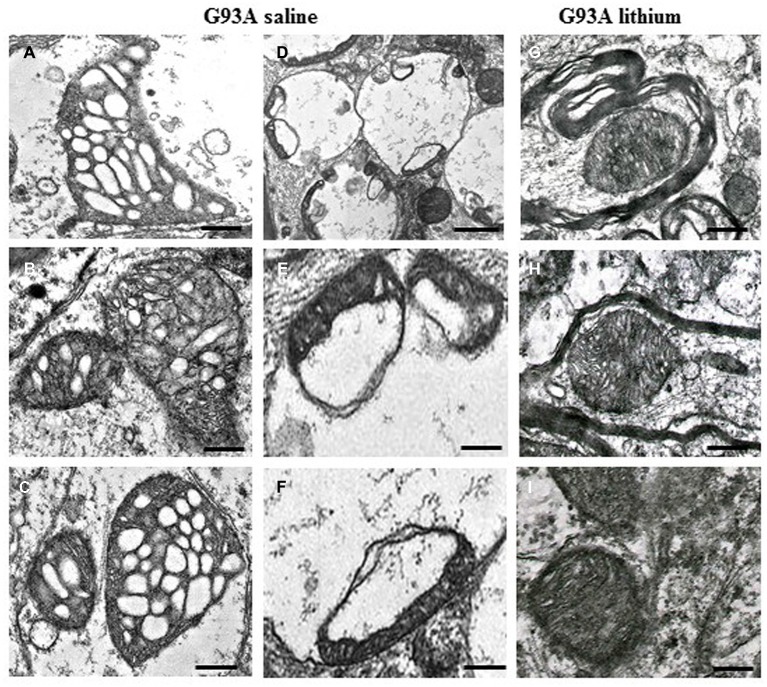
**Paradigm of severe mitochondrial alterations in ALS motor neurons.** The first **(A–C)** and the second column **(D–F)** show at low and high magnification, respectively, the severe damage produced to mitochondria by the SOD1 G93A ALS-inducing mutation. On the right column **(G–I)**, the beneficial effects of autophagy, induced by lithium, are evident. Scale bars: **A–C** = 0.12 μm; **D** = 0.55 μm; **E** = 0.15 μm; **F** = 0.13 μm; **G–I** = 0.12 μm; from Fornai et al. (2008a), Supporting Information, SI Figure 21; Copyright (2008) National Academy of Sciences, USA.

Amyotrophic lateral sclerosis (ALS) is a rapidly progressive neurodegenerative disorder, which is characterized by massive motor neuron loss in the brainstem and spinal cord as well as motor cortex (Charcot, 1874; Boillée et al., 2006). The severity of this neurological disorder led to intense research efforts aimed to elucidate molecular and cellular events underlying motor neuron degeneration. In dissecting the variety of molecular mechanisms which characterize ALS several experimental approaches have been used. Multiple pathways might play a detrimental role on motor neuron survival. In fact, at mitochondrial level the occurrence of altered calcium homeostasis was described in great detail by recent studies (Fuchs et al., 2013; Barrett et al., 2014), while at cellular level the evidence of altered autophagy machinery seems to be well established (Pasquali et al., 2009). Nonetheless, a final common pathway connecting fine molecular mechanisms within mitochondria and pathological events at cellular level still needs to be clarified. Therefore, in the present short manuscript we discuss the significance of ultrastructural evidence, which was established in ALS patients for decades, in connection with altered mechanisms of calcium homeostasis and mitochondrial dynamics. Mitochondrial alterations were described in the ultrastructural pathology of ALS since early 80’s by Atsumi (1981) when analyzing muscle biopsies from ALS patients. Despite their pioneer nature, these studies evidenced the earlier site of mitochondrial alterations at the level of muscle nerve endings. In fact, the routine description of motor neuron cell bodies carried out within ALS spinal cord, despite disclosing some hallmarks of ALS, rules out the potential role of ultrastructural pathology which occurs in motor nerve endings. In keeping with this, some authors emphasized mitochondrial alterations occurring within muscle nerve endings as key mechanisms of disease. Thus, Siklós et al. (1996) pointed out that, at early disease stages, ALS patients develop severe ultrastructural alterations within muscle presynaptic nerve terminals. This is known to consist of increased mitochondrial volume produced by dilution of the matrix and swelling of the organelles featuring broken cristae. These abnormalities represent a hallmark of ultrastructural pathology in ALS where giant mitochondria are often placed within big stagnant vesicular bodies, which were later identified as defective autophagy vacuoles. Remarkably, these findings in ALS patients are replicated by a number of ALS models (Sasaki and Iwata, 1996a,b, 2007; Fornai et al., 2008b; Ferrucci et al., 2010). Therefore, these models provided a useful tool to analyze the neurobiology of disease. For instance, it was established that giant mitochondria are associated with increased neuronal volume (Martin et al., 2007; Fornai et al., 2008a). Again, motor neuron cell body in ALS is filled with giant vesicles (Martin et al., 2007; Fornai et al., 2008a; Laird et al., 2008; see Figure 1). Not surprisingly, these giant vesicles may contain swollen and disrupted mitochondria (Fornai et al., 2008a). These vesicles often fill the whole cell body of motor neurons leading to the concept of slow necrosis (Martin et al., 2007). These vesicles stain for specific autophagy antigens indicating that autophagy pathway is often relented and/or impaired within ALS motor neurons (Fornai et al., 2008a; Laird et al., 2008). The autophagy machinery possesses a specific role in removing altered mitochondria (so-called mitophagy) which suggests that, apart from primary mitochondrial alterations, even a relented removal of aged/altered mitochondria co-exists to produce an overloading of dysfunctional mitochondria within motor neurons.

## The Characterization of Mitochondrial Alterations

Mitochondrial alterations are constantly found within motor neurons of the spinal cord, thus making it mandatory to decipher which molecular mechanism is implicated to comprehend ALS. Seminal studies by a number of research groups clearly demonstrated that mitochondrial alterations are produced by or associate with altered mitochondrial calcium homeostasis. For instance Ladewig et al. (2003) by using multiphoton microscopy and patch clamp recording demonstrated the occurrence of exaggerated calcium release and diminished calcium storage by mitochondria of motor neurons under specific stimuli. This suggests a specific vulnerability of motor neurons to develop disruption of mitochondrial calcium homeostasis upon sustained stimulation. This hypothesis was validated by Jaiswal and Keller (2009) by using a G93A mouse model of ALS. Fuchs et al. (2013) found that during the course of ALS impaired mitochondrial calcium buffering is modified. In detail, in order to compensate for a severe impairment of calcium buffering from spared mitochondria (and the loss of mitochondria) a plasma membrane calcium extrusion mechanism is up-regulated at the end stage of the disease. This suggests an endogenous compensatory mechanism which might be viewed as a promising therapeutic approach to be enhanced by exogenous manipulation. Nonetheless, a recent manuscript by Parone et al. (2013) mitigated and even challenged such a concept.

## A Challenge to the Role of Mitochondria in ALS

Parone et al. (2013) demonstrated that protection of motor neuron mitochondria in the spinal cord induced by inhibiting cyclophilin D (a key regulator of calcium-mediated opening of the mitochondrial transition pore, mTP) in three varieties of superoxide dismutase 1 (SOD1) mutations, despite preserving the number of motor neurons counted in the spinal cord, did neither mitigate symptoms nor prolong survival in experimental ALS. This sharp experimental approach re-introduced the seminal role of peripheral motor denervation as a key determinant in producing palsy and lethality in ALS. These findings lend substance to very early electron microscopy studies in human patients showing that motor axon loss within muscles is advanced at early stages of disease (Atsumi, 1981). Should these data being considered as a challenge to the concept that mitochondria play a pivotal role of in ALS? This is debatable since the occurrence of peripheral degeneration of motor axons is accompanied by severe mitochondrial pathology. Similarly, in their manuscript Parone et al. (2013) did not rule out the detrimental role of mitochondrial alterations. Then, one might consider that mitochondria in ALS motor neuron cell bodies play a sort of epiphenomenal role being not key in disease progression compared with mitochondrial alterations within motor nerve terminals. Similarly, protecting mitochondria within motor neuron cell body does not necessarily relates with protection of mitochondria within motor axons. Thus, being the axonal loss directly responsible for producing palsy and lethality, it is not surprising to observe fatal disease progression in the presence of spared motor neurons counted in the central nervous system. This point of view does not rule out the detrimental effects of mitochondrial alterations but it moves the consequence of mitochondrial damage to which motor neuron compartment is mostly affected. This confirms pioneer studies of Hart et al. (1977) and Hirano et al. (1984a,b) who found the occurrence of altered mitochondria following electron microscopy of motor neurons in patients affected by ALS. Although, it is critical to consider that ultrastructural findings in ALS patients indicate that swollen mitochondria in peripheral nerves occur early than within spinal cord motor neurons (Sasaki and Iwata, 1996a,b, 2007; Siklós et al., 1996).

## How to Reconcile the Altered Mitochondrial Calcium Homeostasis with Previous Point

A very recent manuscript by Barrett et al. (2014) discussed the apparent discrepancy between data obtained with cyclophilin D KO mice and the key role of altered mitochondrial calcium buffering observed in SOD1 mutant mice. These authors provided a series of strong points to reconcile the critical loss of calcium buffering with the lack of protection from symptoms and lethality published by Parone et al. (2013) in cyclophilin D KO mice. For instance the suppression of pathological calcium current observed in cyclophilin D KO mice might not be as effective in axonal mitochondria as that one measured in the motor neuron cell body. This hypothesis includes the chance that axonal mitochondria may possess a kind of calcium alterations which are not preventable by inhibiting expression of cyclophilin D. This includes both a higher variety of mitochondrial stressors within axons compared with cell body and the higher surface-to-volume ratio (loss of the spheroid shape) for axonal mitochondria which would render these organelles richer in density for calcium channels. Similarly, one might add to Barrett et al.’s (2014) considerations that, such a mitochondrial shape would make these organelles more exposed to a toxic microenvironment. When discussing in depth the lack of protection of cyclophilin D KO mice, Barrett et al. (2014) add a number of hypothesis about different mechanisms of neurotoxicity between axons and cell bodies of motor neurons which are plausible indeed. Apart from focusing on differential vulnerability of axon compared with cell body mitochondria, it is worth to be mentioned that a different dynamics may occur for mitochondria placed within motor axons compared with cell bodies. As reported again by Barrett et al. (2014), this difference was first described by Magrané et al. (2012). These authors, by using live imaging microscopy of photo-switchable fluorescent mitochondrial dye, demonstrated that mitochondria from G93A mice possess a slower axonal transport and decreased fusion.

## The Key Role of Mitochondrial Compartments

Altogether, these concepts lead to emphasize the role of motor neuron compartments when considering that mitochondrial alterations do represent a key event in ALS pathogenesis. Therefore, apart from the specific mechanisms it is very likely that the threshold for damage at axonal mitochondria is likely to be lower when compared with the threshold which is needed to damage mitochondria placed in the cell body of motor neurons. This would reconcile the occurrence of axonal denervation in the presence of sparing motor neuron cell bodies described by Parone et al. (2013). Thus, if one analyze the role of mitochondrial dynamics beyond the findings of Magrané et al. (2012, 2014), it is worth to be mentioned that axonal transport it is regulated by the very same class of proteins which regulate autophagy (Pasquali et al., 2014). In fact, altered mitochondrial dynamics should be viewed in a wider perspective where impaired removal of altered mitochondria (impaired mitophagy, which is a part of the autophagy machinery) plays a key role. This impairment indeed occurs in G93A mice (Fornai et al., 2008b; Pasquali et al., 2009) but it seems to extend to other ALS model and ALS related genes (Laird et al., 2008). Similarly, the impairment in mitochondrial dynamics ranges from G93A to TAR DNA binding protein 43 (TDP-43) mutant mice (Magrané et al., 2012, 2014). At the same time, apart from the formal description of a defective mitochondrial fusion (Magrané et al., 2012, 2014), one might extend the analysis to the authentic biogenesis of mitochondria which is defective again in ALS models as shown by polymerase chain reaction (PCR) of mitochondrial genes and MitoTracker green and red (Fornai et al., 2008a). As we shall see in the next paragraph, there is now abundant and very recent evidence, that autophagy of mitochondria is co-activated with mitochondria biogenesis and a defect in autophagy eventually involves a deficiency in mitochondriogenesis, whereas a stimulation of mitophagy concomitantly promotes the biogenesis of novel mitochondria. The mitochondrial compartment then plays a pivotal role in this scenario, where remote axon terminals are expected to be much more affected than neuronal cell bodies.

## Where Damaged Mitochondria Come From?

**Figure 2:**
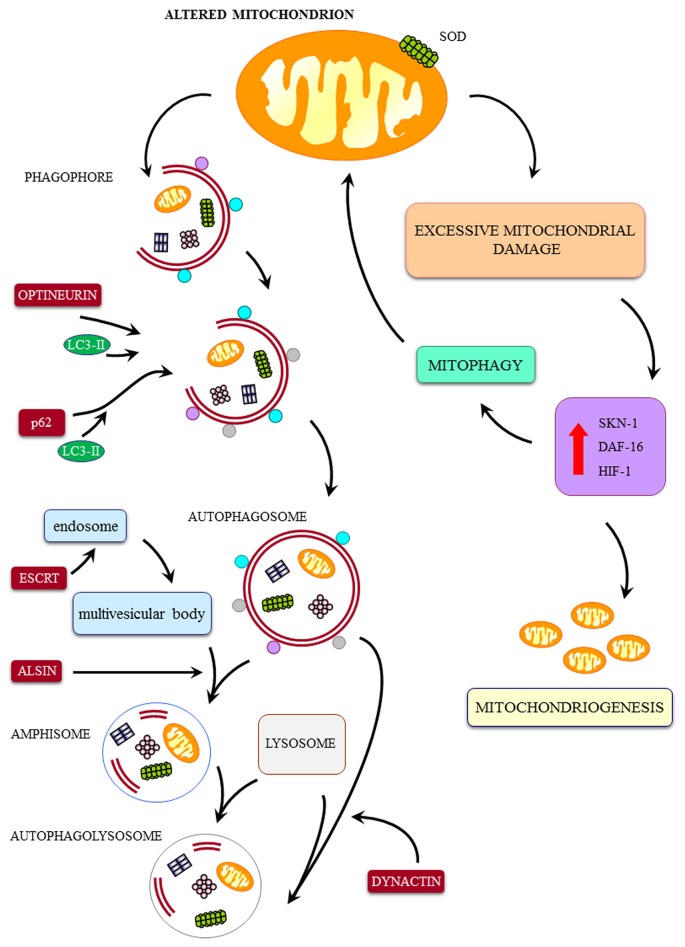
**Cartoon on the major pathways involved in mitochondrial integrity and a few examples of ALS-related alterations.** The mitochondrial dysfunctions in ALS may be produced by a direct mitochondrial toxicity (exemplified here by SOD1-induced mitochondrial toxicity) or a defect in the removal of altered mitochondria by the autophagy/mitophagy pathway. These include: (1) defect in the merging of autophagosome with lysosome (dynactin mutation); (2) defect of merging of endosome with autophagosome to produce amphisome (alsin mutation); (3) defect in linking ubiquitinated protein aggregates to the autophagy machinery by the autophagy protein p62 (SQSTM1 mutation); (4) defect of the fusion of autophagosomes with endosomes and lysosomes (CHMP2B mutation); (5) defect in vesicles trafficking beyond the autophagosome (dynactin mutation); (6) defect in parkin-mediated mitophagy (Optineurin mutation); (7) defect in autophagosome maturation and mitophagy (VCP mutation); and (8) defect in trafficking of autophagy compartments (C9orf72 mutation). Despite a sole defect in the biogenesis of mitochondria may potentially lead to accumulation of degenerated mitochondria, to our knowledge a specific familial ALS (fALS) phenotype due to such a defect was not described so far. Nonetheless, it is likely that, due to a dual tightened control of mitochondrial removal and biogenesis of mitochondria, a failure in the first pathway will eventually lead to a failure in the biogenesis of novel mitochondria. Thus, it is not surprising that, in all fALS phenotypes featuring a defect in the progression of autophagy, we can detect only giant, altered mitochondria in the absence of small, newly synthesized mitochondria. This confirms the eventual concomitance of mitophagy and mitochondriogenesis as indicated by Palikaras et al. (2015a,b). Degenerated mitochondria, to our knowledge a specific fALS phenotype due to such a defect was not described so far. Nonetheless, it is likely that, due to a dual tightened control of mitochondrial removal and biogenesis of mitochondria, a failure in the first pathway will eventually lead to a failure in the biogenesis of novel mitochondria. Thus, it is not surprising that, in all fALS phenotypes featuring a defect in the progression of autophagy, we can detect only giant, altered mitochondria in the absence of small, newly synthesized mitochondria. This confirms the eventual concomitance of mitophagy and mitochondriogenesis as indicated by Palikaras et al. (2015a,b).

When mitochondrial alterations play a pivotal role, than it should be considered whether these may occur directly as the effect of a primary toxicity to mitochondria affecting calcium homeostasis or they can be produced by a defect of mitochondrial removal or even by a relented biogenesis of novel mitochondria. Even in these latter cases abnormal mitochondria are expected to possess altered calcium storage as shown by von Lewinski and Keller (2005). In this scenario several ALS phenotypes are likely to be included (see Figure 2). In fact, in the case of a mutation of the SOD1 gene, an overactive enzyme impairing mitochondrial function is produced (Higgins et al., 2002; Vehviläinen et al., 2014). In addition, in the very same strain of mice an impaired removal of mitochondria due to impaired mitophagy is documented (Pasquali et al., 2009, 2014). This may take a prominent role when specific ALS related proteins are mutated. For instance, the dynactin mutation (Münch et al., 2004) produces a defect in the autophagy flux which in turn is accompanied by stagnant autophagy vacuoles (Laird et al., 2008; Ikenaka et al., 2013). Interestingly, when alterations in the autophagy (mitophagy) machinery are described these are concomitant with defects in the biogenesis of novel mitochondria. In fact, autophagy inducers are described to increase mitochondriogenesis (Struewing et al., 2007; Fornai et al., 2008a), while a common pathway simulates both mitophagy and mitochondriogenesis (Palikaras et al., 2015a,b). Recent data show that mitophagy is tightly related to the biogenesis of novel mitochondria (Palikaras et al., 2015a,b). In detail, when a certain amount of damaged mitochondria is produced, this triggers mitophagy which mediates the removal of damaged mitochondria. This is based on SKN-1 activation, which beside promoting mitophagy, also increases mitochondrial biogenesis (Palikaras et al., 2015a,b). Thus, it is expected that a failure in the autophagy pathway comes together with a defect in the biogenesis of mitochondria. For instance, Palikaras et al. (2015b) hypothesized that suppression of mitophagy inhibits both mitochondria removal and mitochondria biogenesis, thus producing a bidirectional mechanism to increase mitochondrial alterations. Similarly, it is not surprising that autophagy inducers such as lithium or resveratrol, which are autophagy inducers, concomitantly stimulate the biogenesis of novel mitochondria (Fornai et al., 2008a; Meira-Martins et al., 2015; Figure 1). Thus, a sort of tightened dual feedback may adjust mitochondrial population. Not surprisingly, both lithium and resveratrol were found to improve experimental ALS and other motor neuron disorders (Shimada et al., 2012; Mancuso et al., 2014) and synergistic effects in ALS patients are produced by combined administration of autophagy inducers such as valproate and lithium (Boll et al., 2014). However, it is true that the sole increase in the biogenesis of mitochondria does not guarantee for neuroprotection in ALS as shown by Da Cruz et al. (2012). At the same time when autophagy is not induced (Pizzasegola et al., 2009) due to a ten-fold sub-therapeutic treatment (Chiu et al., 2013), the neuroprotective effects induced by lithium on motor neurons cannot be appreciated.

## The Close Connection Between Autophagy and Mitochondria

When focusing on mitochondrial alterations in human ALS, it becomes mandatory to analyze the autophagy status since the occurrence of mitochondrial alterations is likely to be accompanied by a derangement in the autophagy machinery. Confirming this novel standpoint there is evidence in ALS patients that adds on structural mitochondrial alterations showing that a variety of autophagy markers are altered in the spinal cord of ALS patients (Sasaki, 2011). These data often led to opposite interpretation either being considered as a proof for a detrimental role of autophagy in ALS or *vice versa* they have been considered an evidence that a failure of the autophagy machinery occurs in ALS. In keeping with mitochondrial dynamics, it is worth to be mentioned that occurrence of big autophagy vacuoles containing mitochondria generally reflect a defect in the autophagy flux rather than a pathological over-activation of the autophagy machinery. In keeping with this, most familial ALS (fALS) are related with a defect of proteins involved in the autophagy machinery, thereby inducing a failure in the autophagy pathway. A synthetic report of these mutations is reported below along with evidence of a defect in the autophagy machinery. This summarizes and up-dates what already reported by Pasquali et al. (2014).

## A Few Examples of Specific Effects of Human ALS Genes on the Autophagy Machinery

Briefly, more than twenty years ago the SOD1 was the first gene which was associated with fALS (Deng et al., 1993; Rosen et al., 1993). Remarkably, the mutant forms of the SOD1 protein, as well as the wild-type SOD1, are degraded by the autophagy pathway, which in turn, plays a pivotal role in decreasing SOD1 toxicity (Kabuta et al., 2006). In motor neurons from fALS (SOD1) patients and transgenic SOD1 mice as well, autophagy appears to be engulfed by an excess of SOD1. In these cells, a compensatory increase in autophagy markers such as levels of LC3-II occurs (Morimoto et al., 2007; Fornai et al., 2008a), nonetheless, autophagy progression is impaired. This explains why in the presence of SOD1 G93A mutation impairment of autophagy is concomitant with an increase in autophagy-related proteins. The gene ALS2 is responsible for an autosomal recessive fALS (Yang et al., 2001). This gene codes for the alsin protein, which sustains autophagy progression by merging endosomes with autophagosomes to produce amphisomes. In fact, alsin deficiency decreases the motility of endosomes, which accumulate as Rab5 positive giant organelles (Lai et al., 2009). Missense mutations in charged multivesicular protein 2B (CHMP2B) were recently identified in fALS patients (Parkinson et al., 2006). CHMP2B is a component of endosomal sorting complexes required for transport III (ESCRT-III), which belongs to the ESCRT proteins involved in sorting of endocytosed ubiquitinated integral membrane proteins into multivesicular bodies (MVB; Babst et al., 1998, 2002; Katzmann et al., 2001). In particular, CHMP2B enables merging of autophagosomes with either endosomes or lysosomes (Rusten and Stenmark, 2009; Manil-Ségalen et al., 2014). Thus, mutations of CHMP2B lead to impairment in autophagy progression with accumulation of LC3-II positive autophagosomes and altered cargos degradation (Filimonenko et al., 2007; Lee et al., 2007; Cox et al., 2010). The TDP-43 is mostly placed in the nucleus of healthy cells and it is involved in gene transcription and alternative splicing. Patients with TDP-43 mutations develop fALS (Kühnlein et al., 2008; Sreedharan et al., 2008; Van Deerlin et al., 2008; Yokoseki et al., 2008) and possess a misplacement of TDP-43 (from nucleus to cytoplasm) in the form of neuronal inclusions (Arai et al., 2006; Neumann et al., 2006). TDP-43 metabolism is impaired by autophagy inhibitors which produce misplacement of TDP-43, while this is reversed under the effects of autophagy activation (Wang et al., 2010). In line with this, valproate attenuates neuronal toxicity by enhancing autophagy (Wang et al., 2015), while high levels of fragments from TDP-43 engulf the autophagy machinery causing motor deficits (Caccamo et al., 2015). Some fALS patients feature mutations of sequestosome 1 (SQSTM1; Fecto et al., 2011; Rubino et al., 2012). The SQSTM1 gene codes for the protein p62, which is a major autophagy inducer. The specific role of p62 in autophagy consists in linking ubiquitinated protein aggregates to the autophagy machinery (Gal et al., 2007). Heterozygous missense mutations of the dynactin 1 (DCTN1) gene were detected in other fALS patients (Münch et al., 2004). Dynactin mutations produce an autophagy failure (Laird et al., 2008). In fact, dynactin is part of a cytoskeletal molecular complex (consisting of dyenin, dynactin and dynamitin), which is key in promoting the cytoplasmic transport of vesicles along the axon and cell body (Gill et al., 1991; Schroer and Sheetz, 1991; Waterman-Storer et al., 1997). This extends to trafficking of autophagy vesicles such as the merging of autophagosomes with lysosomes (Gill et al., 1991; Schroer and Sheetz, 1991; Waterman-Storer et al., 1997; Laird et al., 2008). In fact, autophagosome needs to be transported along microtubules to the center of the cells (centrosome), where most of the lysosomes are located (Gill et al., 1991; Schroer and Sheetz, 1991; Waterman-Storer et al., 1997). Remarkably, this fALS-producing mutation is a paradigm to connect impairment of autophagy with a compartment-dependent alteration in the flux of organelles (including mitochondria).

Similarly, mutations of optineurin, a protein involved in intracellular trafficking (Ying and Yue, 2012), were described in fALS patients (Maruyama et al., 2010). Optineurin works as an autophagy receptor containing LC3 and ubiquitin-binding domain and it plays a pivotal role in parkin-mediated mitophagy (Wild et al., 2011). Remarkably, optineurin recruits LC3 and clusters around damaged mitochondria upstream to their entrapment within autophagosomes following their parkin-dependent ubiquitination. Thus, it is expected that mutation of optineurin leads to accumulation of damaged mitochondria. Ubiquilin 2, a member of the ubiquilin family, which delivers substrate to autophagy, was found to produce fALS (Deng et al., 2011; Williams et al., 2012). In particular, the loss of ubiquilin inhibits conversion of LC3-I to active lapidated LC3-II, which activates autophagy (Ko et al., 2004; Rothenberg et al., 2010). Mutations of the valosin-containing protein (VCP) gene were described in fALS (Johnson et al., 2010). This gene codes for a chaperone protein involved in mitophagy through autophagosome maturation (Tanaka et al., 2010; Meyer et al., 2012; Yamanaka et al., 2012). Hexanucleotide (GGGGCC) repeat expansions in a non-coding region of chromosome 9 open reading frame 72 (C9orf72) occur in fALS (DeJesus-Hernandez et al., 2011). Very recently C9orf72 was described to be involved in the trafficking of autophagy vesicles (Farg et al., 2014).

## Conclusion

The bulk of mutations reported in the last paragraph, characterize most fALS and indicate a mechanistic connection between autophagy impairment and ALS. This evidence is based on multidisciplinary approaches encompassing *in vitro* protein assay and *in vivo* genetic manipulation. Since the autophagy machinery is key for removing altered mitochondria, it is not surprising that despite a plethora of different mutated proteins in various fALS patients, ultrastructural evidence consistently report the occurrence of a number of altered mitochondria. At the same time, the chronic reiteration of a primary injury towards mitochondria is expected to overwhelm the compensatory mitochondria turn-over. Thus, recent evidence showing impairment of the autophagy machinery in ALS is complementary with the seminal findings showing altered mitochondrial calcium homeostasis in ALS motor neurons. In fact this may occur either as a primary defect or as the consequence of altered mitochondrial turn over. Very recently, such a scenario was remarkably enriched by the evidence that impaired mitophagy necessarily triggers a failure in the biogenesis of novel mitochondria. Thus, the occurrence in ALS of a variety of defects such as: (i) fine mitochondrial dysfunctions, mostly related to calcium homeostasis; (ii) impairment of mitophagy flux; and (iii) failure of mitochondrial biogenesis (often reported as a mere fusion defect) appear more and more as different perspectives to describe similar phenomena.

## Conflict of Interest Statement

The authors declare that the research was conducted in the absence of any commercial or financial relationships that could be construed as a potential conflict of interest.
